# A sub-100 nm thickness flat jet for extreme ultraviolet to soft X-ray absorption spectroscopy

**DOI:** 10.1107/S1600577524001875

**Published:** 2024-04-09

**Authors:** Dario De Angelis, Luca Longetti, Gabriele Bonano, Jacopo Stefano Pelli Cresi, Laura Foglia, Matteo Pancaldi, Flavio Capotondi, Emanuele Pedersoli, Filippo Bencivenga, Marija Krstulovic, Ralf Hendrik Menk, Sergio D’Addato, Stefano Orlando, Monica de Simone, Rebecca A. Ingle, Davide Bleiner, Marcello Coreno, Emiliano Principi, Majed Chergui, Claudio Masciovecchio, Riccardo Mincigrucci

**Affiliations:** a Elettra-Sincrotrone Trieste, Strada Statale 14-km 163.5, Basovizza, 34149 Trieste, Italy; b CNR – Istituto Officina dei Materiali (IOM), Basovizza, Area Science Park, 34149 Trieste, Italy; c Lausanne Centre for Ultrafast Science, Ecole Polytechnique Fédérale de Lausanne, CH-1015 Lausanne, Switzerland; d Laboratory of Atmospheric Chemistry, Paul Scherrer Institut, CH-5232 Villigen PSI, Switzerland; e Dipartimento FIM, Università degli Studi di Modena e Reggio Emilia, Via Campi 213/a, 41125 Modena, Italy; f ISM-CNR, Trieste Branch, in Basovizza Area Science Park, 34149 Trieste, Italy; g Sezione di Trieste, Istituto Nazionale di Fisica Nucleare, Via Valerio 2, 34127 Trieste, Italy; h Department of Computer and Electrical Engineering, Midsweden University, Sundsvall, Sweden; i Department of Chemistry, University College London, 20 Gordon Street, London WC1H 0AJ, United Kingdom; j Laboratory for Advanced Analytical Technologies, EMPA, Uberlandstrasse 129, CH-8600 Dübendorf, Switzerland; Advanced Photon Source, USA

**Keywords:** liquid flat jets, Elettra synchrotron, FERMI free-electron laser, extreme ultra-violet, high-vacuum environments, X-ray absorption spectroscopy

## Abstract

Experimental characterization of the structural, electronic and dynamic properties of dilute systems in aqueous solvents, such as nanoparticles, molecules and proteins, are nowadays an open challenge. X-ray absorption spectroscopy (XAS) is probably one of the most established approaches to this aim as it is element-specific. However, typical dilute systems of interest are often composed of light elements that require extreme-ultraviolet to soft X-ray photons. In this spectral regime, water and other solvents are rather opaque, thus demanding radical reduction of the solvent volume and removal of the liquid to minimize background absorption. Here, we present an experimental endstation designed to operate a liquid flat jet of sub-micrometre thickness in a vacuum environment compatible with extreme ultraviolet/soft XAS measurements in transmission geometry. The apparatus developed can be easily connected to synchrotron and free-electron-laser user-facility beamlines dedicated to XAS experiments. The conditions for stable generation and control of the liquid flat jet are analyzed and discussed. Preliminary soft XAS measurements on some test solutions are shown.

## Abstract 2

The commissioning of the liquid flat jet setup now available at the Elettra synchrotron and FERMI free-electron laser is reported. The setup will allow the user to perform (time resolved-)spectroscopy measurements in liquid environments in almost any open-end beamline at Elettra and FERMI.

## 1. Introduction

Extreme-ultraviolet (EUV) and soft X-ray spectroscopies have demonstrated their power over the years as analytical tools of the chemical and geometric structure of molecular, biological and nano-systems. The remarkable chemical selectivity of X-ray absorption spectroscopy (XAS), targeting specific atoms in multi-element samples, is essential for analyzing complex molecular systems and polyatomic materials, especially if diluted in a solvent.

Time-resolved X-ray spectroscopies have extended chemical selectivity into the time domain for investigations, for example, of short-lived transient species in chemical reactions (Chergui, 2015 ▸; Zhang & Gaffney, 2015 ▸; Chergui & Collet, 2017 ▸; Kraus *et al.*, 2018 ▸) and energy-relaxation pathways in biological systems (Kinschel *et al.*, 2020 ▸; Sension, 2020 ▸; Bacellar & Chergui, 2022 ▸). The advent of high harmonic generation (HHG) sources and free-electron lasers (FELs), which deliver energy-tunable ultrashort pulses, has been exploited to investigate ultrafast dynamics by means of a pump–probe approach (Arrell *et al.*, 2017 ▸; Bergmann *et al.*, 2021 ▸). FELs are nowadays effectively used in this sense, on solid and on liquid samples, both in the destructive and in the non-destructive pump fluence regimes (Smith *et al.*, 2020 ▸; Kleine *et al.*, 2019 ▸; Hejazian *et al.*, 2021 ▸; Chapman, 2019 ▸).

However, the preparation/delivery of samples consisting of complex organic molecules at the probing beam interaction region remains a critical issue. One approach is to deposit a thin layer of the chemical composite on a membrane (Shari’ati & Vura-Weis, 2021 ▸), eliminating the solvent, but this can alter both the structure and the vibrational dynamics of the sample (Waters *et al.*, 2015 ▸; Tao *et al.*, 2010 ▸).

Probing the sample in its liquid environment is often essential and, in this respect, increasing efforts have been made. Hard XFEL facilities nowadays offer a number of available liquid jet delivery systems (Schulz *et al.*, 2019 ▸; Steinke *et al.*, 2016 ▸; Hoffman, Van Driel *et al.*, 2022 ▸; Lima *et al.*, 2023 ▸; Vakili *et al.*, 2022 ▸; Katayama *et al.*, 2019 ▸). These delivery systems, however, cannot be used for experiments in transmission geometry with photons in the EUV and soft X-ray regions because of the high absorbance of solvents at these energies. A first approach to achieve a suitable absorbance consisted of using liquid-containing cells made of silicon carbide (SiC) and/or silicon nitride (Si_3_N_4_) micrometre-thick windows for absorption. The drawback is a large absorption background due to the cell itself.

A free-flowing liquid sample with sub-micrometre thickness was generated for the first time by Ekimova *et al.* (2015 ▸) and was optimized by Fondell *et al.* (2017 ▸) by means of two colliding cylindrical jets. It was exploited for time-resolved XAS with soft X-rays (Smith *et al.*, 2020 ▸), as well as for photoelectron spectroscopy (PES) experiments to investigate liquid–liquid interfaces of heterogeneous solutions (Stemer *et al.*, 2023 ▸).

More recently, flat jets with micrometre and sub-micrometre thicknesses have been generated also via a single converging nozzle (Galinis *et al.*, 2017 ▸; Hoffman, Van Driel *et al.*, 2022 ▸; Crissman *et al.*, 2022 ▸) with a flow-independent thickness. However, this novel jet concept requires a fairly high liquid flow rate (2–10 ml min^−1^), which is suboptimal for scarce samples. Additionally, this jet configuration is typically prone to instabilities and difficulties related to the alignment of the jet. For these reasons, gas-dynamic systems, based on microfluidic chips, have been proposed for the generation of sub-micrometre-thickness liquid sheets with reduced liquid flow (<0.5 ml min^−1^) (Koralek *et al.*, 2018 ▸). Thanks to a properly tuned thickness, and therefore absorbance, the generated liquid foil was exploited in time-resolved XAS experiments (Zhang *et al.*, 2019 ▸) and for ultrafast electron diffraction (Ledbetter *et al.*, 2020 ▸; Yang *et al.*, 2021 ▸). The same chip, using liquids instead of a gas, has been also used for studying the behaviour of liquid–liquid interfaces (Hoffman, Bechtel *et al.*, 2022 ▸; Schewe *et al.*, 2022 ▸).

Recent work highlighted how, for PES on liquids, the cross-sectional area of flat liquid sheets is much larger than that of a cylindrical jet (typical diameter 20 µm), reporting a significant enhancement of both PES signal and liquid-to-gas signal ratio (Yamamoto *et al.*, 2023 ▸). This increased aspect-ratio of the flat jet is also beneficial for transmission experiments, from the perspective of time-resolved experiments, where a thin sample is required to preserve the femtosecond time resolution. Moreover, a planar jet surface was recently exploited for photoelectron circular dichroism (PECD) measurements on solvated species (Malerz *et al.*, 2022 ▸). Furthermore, the flat and thin shape of flat jets is also explored for HHG from liquids (Luu *et al.*, 2018 ▸).

Here, we report on a setup to generate flat liquid jets with a gas-dynamic microfluidic chip. We characterize the jet dimensions as a function of gas and liquid pressures and we report the optimal working parameters in vacuum. The capabilities for EUV and soft XAS in transmission configuration are demonstrated by presenting X-ray absorption spectra of molecular compounds in aqueous solutions. The same setup can also find applications for PES, HHG and other studies where specific thicknesses, aspect ratio and fluid dynamics conditions are required.

## 2. Setup

**Figure 1:**
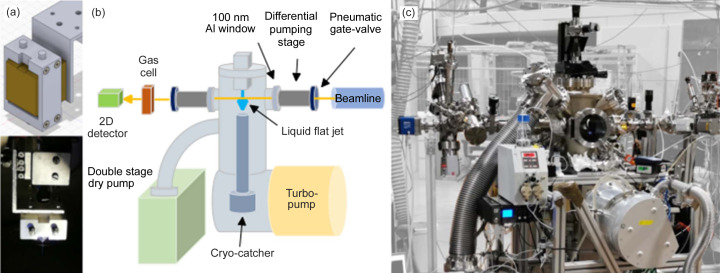
(*a*) Scheme (top) and photograph (bottom) of the custom-made chip holder. (*b*) Scheme of the vacuum setup in the configuration adopted for the FERMI and Elettra beam times. (*c*) Setup installed at the CiPo beamline.

The flat liquid jet setup is sketched in Fig. 1 ▸ and is built around a commercially available borosilicate microfluidics chip from Micronit Microtechnologies BV. A detailed characterization of this gas-dynamic design was given by Hoffman, Bechtel *et al.* (2022 ▸) and Chang *et al.* (2022 ▸). The chip is fabricated by standard lithographic methods to have three microfluidic channels and is optimized to produce a leaf-shaped flat liquid jet by the interaction of three incident jets aligned on a plane: the central 20 µm channel is used for the liquid and the two side 50 µm channels for gas. The gas jets collide symmetrically on the cylindrical liquid jet at a ±40° angle with respect to the latter. The pressure generated by the gas on the liquid forces the cylindrical jet to widen in a confined region, generating a flat sheet of liquid whose thickness depends on both liquid and gas fluxes. In our setup, the liquid flux is controlled by a two-piston pump commonly used in high-performance liquid chromatography (HPLC), whereas the gas flux is directly tuned by a programmable pressure-controlled pump (Elveflow OB1).

The in-house manufactured chip holder [Fig. 1 ▸(*a*)] includes standard microfluidic connectors for the liquid and gas feeding to the nozzle and is mounted on a four degrees-of-freedom manipulator into the dedicated vacuum chamber [Figs. 1 ▸(*b*) and 1 ▸(*c*)]. A three-axes piezoelectric translator by Smaract between the holder and the manipulator was used to fine-tune the position of the jet. A first coarse alignment of the chip is achieved with the manual manipulator and fine alignment, as well as the transverse scans, are made with the Smaract piezoelectric stages. The entire liquid jet setup is conceived to work independently and to be potentially installed on any open-port beamline. For this reason, we performed the preliminary operational tests on the self-standing chamber. To sustain the pressure load of gas flowing through the dynamical nozzles, the vacuum vessel was equipped with a 1600 l min^−1^ turbomolecular pump and a 1000 l min^−1^ two-stage dry pump. Both vacuum pumps were insulated by the main chamber by the front pneumatic valves. In order to collect and freeze the liquid debris after the interaction of the flat jet with the EUV/soft X-ray beam, a cryogenic reservoir was installed in the vacuum chamber a few centimetres below the chip.

Under operational conditions, the pressure inside the chamber spans from 10^−3^ mbar to 10^−2^ mbar, depending on the liquid and gas fluxes, which finally determine the flat jet shape. Indeed, stable sample delivery conditions are achieved when the liquid stream is well directed towards the cryogenic trap. We observed that a high gas pressure produces a thinner liquid foil, but it also induces droplet formation with consequent dispersion of liquid debris outside of the cryogenic reservoir entrance, which therefore provokes a pressure rise above 10^−2^ mbar in the chamber. A compromise between foil thickness and chamber pressure is therefore necessary. Moreover, unstable conditions can arise from extreme liquid pressures, eventually resulting in jet freezing, which forces a stop of the system operation.

The experimental setup was designed to perform XAS measurements in transmission mode using a PCO sCMOS cooled detector (Menk *et al.*, 2022 ▸), adapted for high-vacuum operation and installed downstream of the interaction region [Fig. 1 ▸(*b*)] on a differentially pumped stage.

Such a 2D detector gives us the possibility to collect projection images of the liquid foil exploiting the collimated photon beam of the synchrotron beamline. In this manner, we are able to irradiate the entire jet and select the pixels in which absorption is measured. In this way, we optimize at the same time the sample illumination and the signal-to-noise ratio (SNR). This remarkable advantage over a traditional photodiode detector comes with almost no additional issues from an instrumental point of view. The detector needs in any case to be mounted on a differential pumping stage to prevent the deposition of solution on top of it.

A Basler camera, equipped with a standard photographic objective, was installed outside the chamber for monitoring the nozzle in real time through a view port.

The liquid jet vacuum chamber was decoupled from the beamline and detector high-vacuum environments using 100 nm-thick aluminium foils mounted on windowed ConFlat manual gate valves. The material of these thin windows was selected to reach the best compromise between EUV transparency and gas permeability. Aluminium is commonly used to filter out spurious visible radiation contained in the FEL pulses, so in our case it does not introduce any criticality on the EUV beam transport. On the other hand, the aluminium foils we used showed some microporosity, which prevented the pressure in the differential pumping stages from dropping below 10^−5^ mbar under operating conditions. Possible reactivity with chemical species contained either in the liquid or in the solute was also considered. Aluminium proved to be a valuable choice in our case. Additional manual gate-valves were installed close to the windowed ones for the pumping and venting procedure. A pneumatic valve was installed between the differential pumping section and the beamline port. The valve controller was connected to a pressure gauge so that a rapid pressure increase in the differential pumping section, due to possible Al foil breakage, would have caused the closure of the pneumatic valve, thus preventing liquid and gas backflow to the beamline/detectors.

The flat jet was started at ambient pressure in its cylindrical configuration with the liquid flux parameters already set at the standard operation values; the gas flow for jet flattening was activated under vacuum conditions. At the starting phase, the beamline, detector and two differential pumping stages were under vacuum, with the manual gate valves closed and windowed valves open. Once the pressure in the liquid jet chamber was about 10^−2^ mbar, the windowed valves were closed and the manual gate valves were opened. The same operation was repeated backwards for venting.

## 3. Structural characterization of the jet

**Figure 2:**
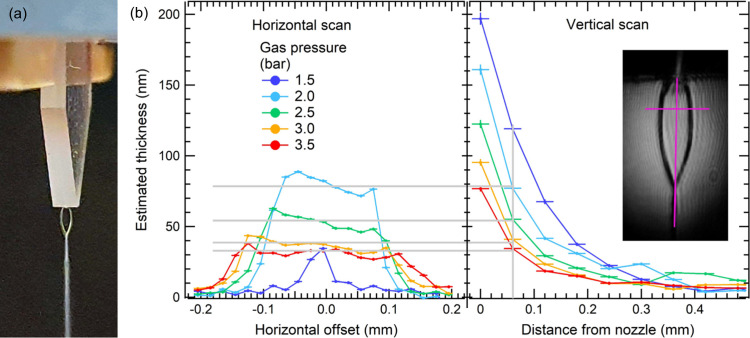
(*a*) Photograph of the flat jet under operation conditions inside the experimental chamber. (*b*) Thickness profiles calculated from the transmission measurement at 54.4 eV acquired by scanning the flat jet horizontally and vertically; each profile has been acquired at different gas pressures, keeping the liquid flux constant. Inset: a photograph of the flat jet operated at 3.5 bar gas pressure, acquired with a visible camera. Scan paths are highlighted in magenta.

As part of the commissioning activity, we performed X-ray absorption measurements at the DiProI beamline at FERMI (Capotondi *et al.*, 2013 ▸, 2015 ▸) to characterize the flat liquid jet. The experimental setup was installed downstream of the main experimental chamber of the beamline and the FEL beam was focused on the jet position by means of adaptive bendable Kirkpatrick–Baez mirrors (Raimondi *et al.*, 2013 ▸). The FEL spot size on the jet was set to 30 µm × 30 µm (FWHM), *i.e.* considerably smaller than the flat jet surface area. We used high-purity water as a test sample and air for jet-shaping. We measured the X-ray absorption by scanning the flat jet in the horizontal and vertical directions, in correspondence with the maximum width and height of the leaf. The flat jet obtained during this characterization is shown in Fig. 2 ▸(*a*), while the resulting thicknesses of the flat jet measured at 54.4 eV are reported in Fig. 2 ▸(*b*) as a function of the gas pressure. We can easily observe that, for the lowest gas pressure, the shape of the jet does not sensibly differ from the cylindrical one. Under this condition, the section of the FEL beam is larger than the jet diameter and the absorption is underestimated, therefore leading to an unrealistic estimate of about 35 nm at 1500 mbar gas pressure displayed in Fig. 2 ▸(*b*). With our setup, the jet is assumed to have the desired flat shape at gas pressure values above 2 bar, and the flat jet thickness can be calculated from the absorption at 54.4 eV. As shown in Fig. 2 ▸(*b*), the maximum lateral size for the leaf was estimated to be around 200 µm, obtained with 3500 mbar gas pressure (red curve in the graph). Considering the absorption of liquid water at the selected photon energy (Hoffman, Bechtel *et al.*, 2022 ▸), the estimated jet thickness for this limit case is 35 ± 2 nm, in agreement with the measurements performed by Koralek *et al.* (2018 ▸). Stable thickness control can be achieved by modulating the gas pressure that generates the liquid foil. Fig. 2 ▸(*b*) shows that, at about 50 µm from the liquid exit point, the foil thickness can be varied from 35 nm to 80 nm, decreasing the gas pressure from 3500 mbar to 2000 mbar. Consistent with a previous investigation (Koralek *et al.*, 2018 ▸) on similar injection systems, a gradual decrease of liquid sheet thickness is observed by moving the probe beam far from the input point of the liquid stream.

## 4. Spectroscopy measurements

After characterization of the geometric properties of our flat jet setup, an investigation was performed to test its effectiveness for the acquisition of X-ray absorption spectra. The experiment was carried out at the Circular Polarization (CiPo) beamline (Desiderio *et al.*, 1999 ▸) at the Elettra synchrotron, where the installation of a mobile experimental endstation was possible and monochromated radiation in the range 40–1000 eV was available.

We focused on relevant photon energies in the soft X-ray energy range that are strategic for the investigation of organic compounds. Water was used as solvent and two model samples were selected: ferric tris-oxalate [Fe(C_2_O_4_)_3_]^3−^ (CAS: 13268-42-3), hereafter referred to as Fe-OX, and glycine (CAS: 56-40-6).

We used air to feed the gas circuit for all the measurements. Measurements were performed at photon energy ranges of inner shell absorption features of the two solutes. Fe-OX, an ionic compound highly soluble in water, was investigated in the energy ranges 700–720 eV and 520–560 eV at the Fe *L*_2,3_-edge and O *K*-edge, respectively. The glycine solution was probed at the C *K*-edge around the 280–310 eV energy window. The solutions were prepared at concentrations of 1 *M* for glycine and 0.5 *M* for Fe-OX. Under operating conditions, typical sample flow rates were between 0.4 ml min^−1^ and 0.45 ml min^−1^. The maximum lateral size of the jet was set to 0.2 ± 0.01 mm; the estimated thickness, on the basis of the characterization done on DiProI, was 40 nm to 50 nm. For absolute-energy calibration of the XAS measurements, total ion yield (TIY) spectra of calibrant gases (Xe, CO_2_) were acquired by means of a gas cell installed downstream of the sample, between the sCMOS detector and the experimental chamber.

**Figure 3:**
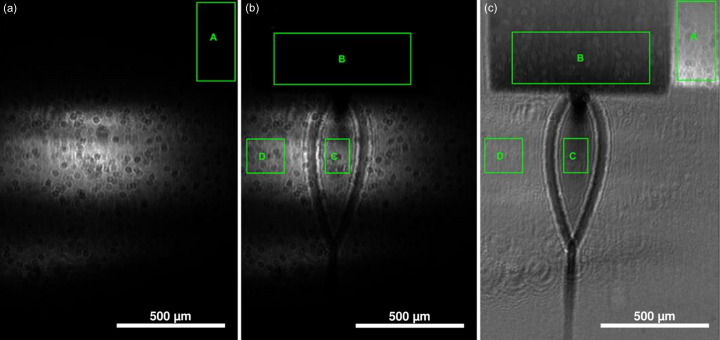
Projection images acquired by the CMOS camera at the end of the beamline endstation. (*a*) Image acquired while the liquid-jet chip was moved out of the beam (sample-out). (*b*) Image acquired with the liquid-jet chip in the measurement position (sample-in). (*c*) Image derived from the previous two as the (sample-in)/(sample-out) ratio. The four ROIs are shown: A and B are the background ROIs for the sample-out and sample-in images, respectively, usually taken in the chip shadow; C is the signal ROI; D is the normalization ROI.

The photon beam was collimated and shaped into a 10 mm × 5 mm rectangular section [visible in Figs. 3 ▸(*a*) and 3 ▸(*b*)] in such a way to illuminate a large part of the flat jet and to produce a clearly identifiable image on the detector. The X-ray flux measured upstream of the liquid jet chamber was about 5 × 10^8^ photons s^−1^.

The data acquisition scheme for each measurement consisted of a sample-in (signal image) and a sample-out acquisition (blank image) of the photon beam only, in order to record the transmitted intensity (*I*) and the incident intensity (*I*_0_), respectively. Blank measurements were performed by completely removing the liquid sheet from the beam path, while keeping gas and liquid streams open. In this way, the same conditions could be attained in both sets of measurements, taking into account the absorption contribution from water vapor. This two-step acquisition procedure allowed us to remove the gas phase contribution from the final absorbance, since it is considered to be the same when measuring both *I* and *I*_0_. At each energy step, the measurement outcome is the sum of 100 to 200 acquired frames, whose exposure time was 20–25 ms per frame (corresponding to 2–5 s integration time per energy point). Fig. 3 ▸ provides more details about the region of interest (ROI) used to extract the absorbance. For each photon energy value, the absorbance value (Abs) was calculated as

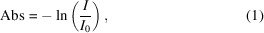

where

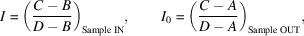

with *A*, *B*, *C* and *D* indicating the average intensity value measured at the corresponding ROI. For both sample-in and sample-out measurements, the background-subtracted intensity in signal ROI *C* was normalized by the intensity in ROI *D*, which samples a portion of the photon beam. This allowed us to estimate *I* and *I*_0_ in a consistent way even in the case of small changes in the experimental conditions (especially in the beam characteristics) between the two measurements.

**Figure 4:**
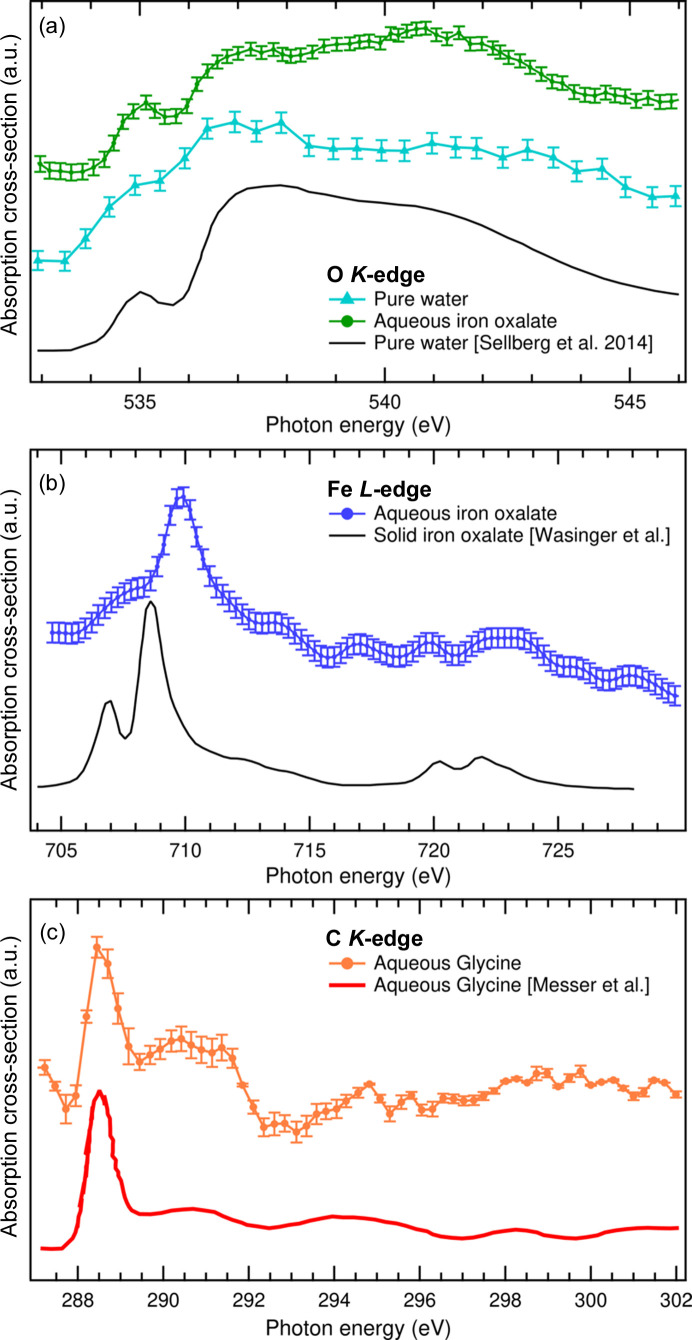
XAS spectra of the sample solutions: (*a*) O *K*-edge of water and iron(III) oxalate solution, (*b*) Fe *L*-edge of iron(III) oxalate solution, (*c*) C *K*-edge of glycine.

Fig. 4 ▸ shows the XAS spectra of the above-mentioned samples. The oxygen *K*-edge was measured with the aim of disentangling the solvent and solute absorption contribution in the case of Fe-OX solution [Fig. 4 ▸(*a*)]. It is possible to recognize the solute contribution by comparing the two post-edge spectral features at 537 eV and 541 eV [see data from Sellberg *et al.* (2014 ▸) in the same plot for a pure water reference]: the intensity difference between these two components has an opposite sign for the Fe-OX solution. This implies that the signal coming from the oxygen atoms of the solute molecules is recognizable. We noted that the tuning of the liquid jet thickness proved to be a key parameter in optimizing the SNR.

The Fe *L*-edge [Fig. 4 ▸(*b*)] was measured with the same jet parameters used for the O *K*-edge. From the comparison with the absorption measurement reported in the literature by Wasinger *et al.* (2003 ▸) – performed on a solid sample – we identified the main feature characterizing the *L*_3_-edge absorption spectrum for Fe-OX. The larger FWHM (1.5 eV instead of 1.1 eV) can be ascribed to the energy resolution of the CiPo beamline.

To avoid photo-degradation of Fe-OX, we wrapped the solution flask in Al foils. Nevertheless, we cannot exclude that an enlargement of the spectral features is due to iron-reduced photoproducts.

On the other hand, the reduced SNR with respect to the solid-state reference can be attributed to lower statistics, which are related to the lower density of Fe atoms in the water solution with respect to the solid sample (about one order of magnitude). Moreover, we measured an edge shift of 1.0 ± 0.2 eV towards higher energies. This effect can be due to the radically different environments between these two samples. A deeper and more detailed explanation of this effect is beyond the scope of this work.

In the investigation of the glycine solution, we focused on the C *K*-edge spectral region, shown in Fig. 4 ▸(*c*). Due to its lower absorption cross-section compared with the Fe *L*-edge absorption, and despite higher glycine concentration, the spectra on C have lower SNR, under the same jet conditions and acquisition statistics. This is due to the significantly lower photon flux at these energies, resulting from the different operating configuration of the beamline insertion device (wiggler versus undulator) and from the carbon-contaminated beamline mirrors. Despite this, the C *K*-edge features stand out in the spectrum [see comparison with literature data acquired in the liquid phase (Messer *et al.*, 2005 ▸)]. In particular, the strong 1*s* → π* resonance on the carb­oxy­lic carbon is well resolved above the signal noise.

For the three spectral regions described above, the data were collected by exposing the camera for 2–5 s at each photon energy, depending on the acquired number of frames. An increase of 10 to 50 times in the acquisition time would be required for a better SNR. The intensity fluctuation visible in the spectra in Fig. 4 ▸ are experimental artifacts due to an unstable photon flux of the synchrotron beamline that were not completely corrected by the beamline *I*_0_ monitor for incidental technical reasons. Nevertheless, from the perspective of potential pump–probe experiments, it would be totally removed with a shot-to-shot differential acquisition scheme (Saes *et al.*, 2004 ▸).

Moreover, we remark that the setup described requires large amounts of solution, which cannot always be available. To address this limitation, a recycling liquid collection system is under development.

## 5. Conclusions

In this work, we describe a new setup consisting of flat liquid jet for steady-state and time-resolved soft X-ray absorption of dilute systems. We demonstrated that a sub-micrometre-thickness flat jet can be achieved with the setup described. Operational conditions have been reported and the flat jet thickness was shown to reach tens of nanometres such that absorption measurements in the EUV region are possible. We also demonstrated how the experimental system can withstand the high gas load due to the flat jet (vapor and gas) and that the chamber was properly decoupled from the surrounding vacuum equipment via Al-foil windows, conditions required to operate in beamlines where an EUV/soft X-ray compliant UHV environment is needed.

Moreover, we demonstrated that the devised setup is suitable for spectroscopic measurements in the energy range at and above the carbon *K*-edge. We reported absorption spectra of solvated species in aqueous solution at the C *K*-edge, O *K*-edge and Fe *L*-edge in transmission configuration. For these three spectral regions, a clear signal from the solvated species is observed, though further acquisition time could be sensibly beneficial for a better SNR. The compatibility of the setup with FEL beamlines appears promising for sub-picosecond dynamical investigation of photo-activated samples. Further setup improvements are under evaluation to achieve better spectroscopic performances and to probe at lower energies, accessing absorption edges of elements in the EUV range, such as the *M*- and *N*-edges of metal atoms. This will pave the way to investigations of further electronic state dynamics, which can be interesting from a chemical point of view. Moreover, further instrumental developments on the TIMEX beamline at FERMI are planned in order to host a liquid jet setup. With this setting, correlation-based spectroscopy measurements can be allowed (De Angelis *et al.*, 2023 ▸), which improve the efficiency of the measurement.

Finally, exploiting the features of the setup presented here, especially the high tuneability of the liquid sample thickness (and consequently absorbance), we plan to perform pump–probe spectroscopy measurements in the visible to EUV/soft X-ray range. In this context, the flowing sample would remove the problem of sample degradation and the jet flatness would provide a sufficiently large interaction volume, limiting, at the same time, the chirp of the light pulse. All this will be beneficial in terms of measurement time and sample consumption, opening a new path for the characterization of sub-picosecond time-scale molecular dynamics.
